# Addition of Y-90 radioembolization increases tumor response and local disease control in hepatocellular carcinoma patients receiving sorafenib

**DOI:** 10.1007/s00259-022-05920-8

**Published:** 2022-08-02

**Authors:** Osman Öcal, Kerstin Schütte, Christoph J. Zech, Christian Loewe, Otto van Delden, Vincent Vandecaveye, Chris Verslype, Bernhard Gebauer, Christian Sengel, Irene Bargellini, Roberto Iezzi, Alexander Philipp, Thomas Berg, Heinz J. Klümpen, Julia Benckert, Maciej Pech, Antonio Gasbarrini, Holger Amthauer, Peter Bartenstein, Bruno Sangro, Peter Malfertheiner, Jens Ricke, Max Seidensticker

**Affiliations:** 1 Department of Radiology, University Hospital, LMU Munich, Marchioninistrasse 15, 81377 Munich, Germany; 2 Department of Internal Medicine and Gastroenterology, Niels-Stensen-Kliniken Marienhospital, Osnabrück, Germany; 3 Klinik für Gastroenterologie, Hepatologie und Endokrinologie, Medizinische Hochschule Hannover (MHH), Carl-Neuberg-Straße 1, 30625 Hannover, Deutschland; 4 Radiology and Nuclear Medicine, University Hospital Basel, University of Basel, Basel, Switzerland; 5 Section of Cardiovascular and Interventional Radiology, Department of Bioimaging and Image-Guided Therapy, Medical University of Vienna, Vienna, Austria; 6 Department of Radiology and Nuclear Medicine, Academic University Medical Centers, Amsterdam, The Netherlands; 7 Department of Radiology, University Hospitals Leuven, Leuven, Belgium; 8 Department of Digestive Oncology, University Hospitals Leuven, Leuven, Belgium; 9 Department of Radiology, Charité – University Medicine Berlin, Berlin, Germany; 10 Radiology Department, Grenoble University Hospital, La Tronche, France; 11 Department of Vascular and Interventional Radiology, University Hospital of Pisa, Pisa, Italy; 12 UOC di Radiologia, Dipartimento di Diagnostica per Immagini, Radioterapia Oncologica ed Ematologia, Fondazione Policlinico Universitario A. Gemelli IRCCS, Rome, Italy; 13 Department of Medicine II, University Hospital, LMU Munich, Munich, Germany; 14 Klinik und Poliklinik für Gastroenterologie, Sektion Hepatologie, Universitätsklinikum Leipzig, Leipzig, Germany; 15 Department of Medical Oncology, Amsterdam University Medical Centers, Amsterdam, the Netherlands; 16 Department of Hepatology and Gastroenterology, Charité – Universitätsmedizin Berlin, Campus Virchow Klinikum, Berlin, Germany; 17 Departments of Radiology and Nuclear Medicine, University of Magdeburg, Magdeburg, Germany; 18 Fondazione Policlinico Universitario Gemelli IRCCS, Universita’ Cattolica del Sacro Cuore, Rome, Italy; 19 Corporate member of Freie Universität Berlin and Humboldt Universität zu Berlin, Department of Nuclear Medicine, Charité – Universitätsmedizin Berlin, Augustenburger Platz 1, 13353 Berlin, Germany; 20 Department of Nuclear Medicine, University Hospital, LMU Munich, Munich, Germany; 21 Liver Unit, Clínica Universidad de Navarra and CIBEREHD, Pamplona, Spain

**Keywords:** Radioembolization, Sorafenib, mRECIST, Objective response, Hepatocellular carcinoma

## Abstract

**Purpose:**

To compare the treatment response and progression-free survival (PFS) in advanced hepatocellular carcinoma (HCC) patients who received sorafenib treatment either alone or combined with radioembolization (RE).

**Methods:**

Follow-up images of the patients treated within a multicenter phase II trial (SORAMIC) were assessed by mRECIST. A total of 177 patients (73 combination arm [RE + sorafenib] and 104 sorafenib arm) were included in this post-hoc analysis. Response and progression characteristics were compared between treatment arms. Survival analyses were done to compare PFS and post-progression survival between treatment arms. Multivariate Cox regression analysis was used to compare survival with factors known to influence PFS in patients with HCC.

**Results:**

The combination arm had significantly higher objective response rate (61.6% vs. 29.8%, *p* < 0.001), complete response rate (13.7% vs. 3.8%, *p* = 0.022), and a trend for higher disease control rate (79.2% vs. 72.1%, *p* = 0.075). Progression was encountered in 116 (65.5%) patients and was more common in the sorafenib arm (75% vs. 52.0%, *p* = 0.001). PFS (median 8.9 vs. 5.4 months, *p* = 0.022) and hepatic PFS were significantly better in the combination arm (9.0 vs. 5.7 months, *p* = 0.014). Multivariate analysis confirmed the treatment arm as an independent predictor of PFS.

**Conclusion:**

In advanced HCC patients receiving sorafenib, combination with RE has an additive anticancer effect on sorafenib treatment resulting in a higher and longer tumor response. However, the enhanced response did not translate into prolonged survival. Better patient selection and superselective treatment could improve outcomes after combination therapy.

## Introduction

Hepatocellular carcinoma (HCC) is the most common primary liver cancer, and in up to 90% of patients, HCC develops in a cirrhotic liver [1]. Approximately 70% of the patients present at stages that preclude potentially curative treatment options [2]. Sorafenib treatment has been shown to improve survival in advanced HCC patients [3, 4]; it has been the standard of care for advanced HCC cases with preserved liver function for over a decade, and with the approval of atezolizumab-bevacizumab combination, it has shifted from first- to second-line [5]. Many non-randomized studies have shown that Yttrium-90 (Y-90) radioembolization (RE) is an effective locoregional treatment option with high tolerability [6–9]. However, two randomized controlled trials have failed to show a survival benefit of RE compared to sorafenib in the first-line setting [10, 11]. Further on, in the SORAMIC trial (SORAfenib in combination with local MICro-therapy guided by gadolinium-EOB-DTPA–enhanced MRI, EudraCT 2009–012576-27, NCT01126645), the combination of RE with sorafenib showed no improved survival compared to sorafenib monotherapy in the first-line [12].

Nevertheless, during the recruitment period of these three trials, no second-line systemic treatment option was available for patients who progressed under sorafenib treatment. During the last few years, further systemic treatment options have been shown to have a survival benefit [13–15]. This condition underlines the importance of secondary outcome parameters other than overall survival for HCC patients recruited in these trials, such as progression-free survival (PFS), objective response, and disease control [16]. Due to unique challenges in imaging assessment of HCC, criteria for evaluation of these imaging-based secondary outcome parameters have been developed, and response analysis by mRECIST has been shown to correlate with survival in HCC patients who underwent locoregional therapies, including RE [17, 18]. Furthermore, the correlation between survival and objective response according to mRECIST after sorafenib treatment has been confirmed in the SILIUS trial and in the post-hoc analysis of sorafenib arm of the SORAMIC trial [19, 20].

Additionally, some modern imaging criteria have been described to identify cancer patients who do not benefit from treatment. Early tumor shrinkage (ETS) and depth of response (DpR) have been shown to correlate with treatment outcome in various tumor types [21, 22].

This post-hoc analysis of the SORAMIC trial aimed to compare objective response rates, progression-free survival, and response characteristics of combination and sorafenib arms according to mRECIST and modern response criteria, including ETS and DpR, with independent imaging review.

## Material and methods

### Study design and patient population

This study is a post-hoc analysis of a subset of the patients from the palliative arm of the SORAMIC trial, a prospective, randomized-controlled phase II trial exploring the additional effect of RE to sorafenib treatment, performed in 38 centers in 12 countries in Europe and Turkey. The inclusion and exclusion criteria for the SORAMIC trial have been described previously [12]. In summary, patients aged 18 to 85 years with advanced HCC, preserved liver function (Child–Pugh scores A to B7), an Eastern Cooperative Oncology Group performance status ≤ 2 were eligible. If the disease was liver-dominant and lungs were not involved, extrahepatic metastases were permitted. The study protocol was approved by the institutional review board and competent authorities, and all patients gave written informed consent. Response assessment and its correlation with survival of the sorafenib arm were previously published [20].

Only the per-protocol (PP) population of the trial was considered in this analysis, and the availability of cross-sectional follow-up images for a centralized review was required for inclusion to this substudy. Exclusion criteria were (1) no follow-up within the 6 months after randomization and (2) a period of more than 6 months without imaging follow-up before death, unless progression was already encountered.

Patients were randomized in an 11:10 ratio to receive either combination of RE and sorafenib or sorafenib monotherapy. In patients randomized into the combination arm, RE was performed with a sequential lobar fashion in patients with bilobar disease or only to the affected lobe in case of unilobar disease, and sorafenib treatment was initiated 3 days after the last RE session. Relevant hepatopulmonary shunt and extrahepatic microsphere accumulation were excluded before RE session. The prescribed activity of resin particles was calculated from the body surface area, the percent tumor involvement in the liver, and the percent of lung shunting. Sorafenib was started at a dose of 200 mg twice daily, and if tolerated, the dose was escalated to 400 mg twice daily after 1 week.

Before recruitment, all patients underwent CT and MRI according to the published protocol of the diagnostic arm of the SORAMIC trial, and follow-up with CT or MRI every 3 months after treatment was recommended [23]. However, follow-up imaging modality was at the discretion of the participating centers, and in some centers, surveillance was done with sonography. At the end of the study, cross-sectional images were requested from each center. Out of 288 patients in the PP population, 222 patients had available follow-up images for central analysis.

Baseline and follow-up images of each patient were reviewed according to mRECIST by a radiologist blinded to the treatment arm and all clinical information. Up to two liver lesions were selected as target lesions in baseline images of each patient (*hepatic target lesions*), according to described criteria [16]. In patients with extrahepatic disease, up to three extrahepatic target lesions with a maximum of 2 lesions per organ were identified. Other lesions were recorded as non-target lesions. Progression-free survival and time-to-progression were censored at last available follow-up images. Besides response evaluation according to mRECIST, early tumor shrinkage (ETS, more than 20% diameter decrease in the enhancing part of *hepatic target lesions* at the first follow-up) was evaluated. Additionally, in patients with disease control, depth of response (DpR), described as the percentage of maximum diameter decrease in arterially enhancing portion, was calculated considering *hepatic target lesions*.

**Fig. 1:**
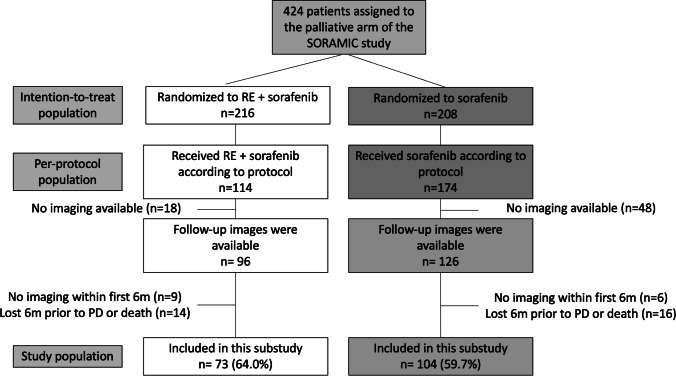
Consort diagram. RE: radioembolization, PD: progressive disease

Forty-five patients were excluded due to previously defined exclusion criteria on the consistency of follow-up, and, finally, 177 patients (61.4% of the PP population) were included in this analysis (Fig. 1).

### Statistical analysis

All statistical analyses were performed using R statistical and computing software, version 3.5.0 (http://www.r-project.org). Categorical variables were reported as counts and percentages, and continuous variables were reported as means and standard deviations. Correlations were evaluated with Chi-square and Fisher’s exact tests, and *t*-test was used to compare two groups. Objective response and disease control rate estimates (including the 95% confidence interval [*CI*]) were calculated using the Clopper-Pearson exact binomial method. The Kaplan–Meier method was used for estimates of overall, progression-free, and post-progression survivals and time-to-progression. Cox regression models were used to assess the effects of cofounding factors on survival. Statistically significant variables in the univariable analyses were analyzed in multivariable Cox regression models to explore prognostic factors of overall survival.

## Results

### Baseline characteristics

A total of 177 patients with a median survival of 14.3 months were included in this study. While 73 patients were in the combination arm of the trial, 104 patients in sorafenib arm. The median number of follow-up images was 3 (interquartile range, 2–5) in the combination arm and 3 (interquartile range, 2–5) in the sorafenib arm. Baseline patient characteristics of both arms are summarized in Table 1. There were more patients with extrahepatic involvement in the combination arm (15% vs. 5.7%, *p* = 0.038), and ALBI grade 2 (54.8% vs. 34.2%, *p* = 0.011) in the sorafenib arm. Except for these parameters, the baseline characteristics of both arms were similar. Overall survival was 15.0 (12.4–19.6) months in the combination arm and 13.8 (10.5–17.5) months in the sorafenib arm. There was no difference in overall survival between study arms (*HR*, 1 [0.76–1.5], *p* = 0.77).

**Table 1:** Patient demographics and comparison of baseline characteristics of patients

	Overall (*n* = 177)	RE + sorafenib (*n* = 73)	Sorafenib (*n* = 104)	*p*
Gender (male)	155 (87.5)	65 (89.0)	90 (86.5)	0.619
Age (≥ 65 years)	89 (50.2)	39 (53.4)	50 (48.1)	0.483
Race (White)	159 (89.8)	67 (91.7)	92 (88.4)	0.471
ECOG				
• 0 • 1 • Missing	134 (75.7) 40 (22.5) 3 (1.6)	52 (71.2) 18 (24.6) 3 (4.1)	82 (78.8) 22 (21.1) 0	0.483
Liver cirrhosis (yes)	153 (86.4)	60 (82.1)	93 (89.4)	0.166
HCC etiology				
• Hepatitis B • Hepatitis C • Alcohol	14 (7.9) 48 (27.1) 83 (46.8)	4 (5.4) 18 (24.6) 36 (49.3)	10 (9.6) 30 (28.8) 47 (45.1)	0.402 0.537 0.588
Previous TACE	45 (25.4)	20 (27.3)	25 (24.0)	0.613
Diffuse disease (≥ 10 lesion)	83 (46.8)	39 (53.4)	54 (51.9)	0.843
Median (mean) target lesion size, mm	59 (65.1)	62 (67.1)	57.5 (63.7)	0.590
Portal vein infiltration	85 (48.0)	30 (41.1)	55 (52.8)	0.122
Extrahepatic spread	17 (9.6)	11 (15.0)	6 (5.7)	**0.038**
Child Pugh				
• A • B	165 (93.2) 12 (6.8)	70 (95.9) 3 (4.1)	95 (91.3) 9 (8.7)	0.363
BCLC				
• A/B • C	51 (28.8) 126 (71.2)	22 (30.1) 51 (69.8)	29 (27.9) 75 (72.1)	0.744
Up to 7 (yes)	148 (83.6)	60 (82.1)	88 (84.6)	0.668
ALBI-grade				
• 1 • 2 • Missing	91 (51.4) 82 (78.8) 4 (3.8)	45 (61.6) 25 (34.2) 3 (4.1)	46 (44.2) 57 (54.8) 1 (0.9)	**0.011**
AFP > 400	55 (31.1)	23 (31.5)	32 (30.7)	0.890

*AFP*, alfa-fetoprotein; *ALBI*, albumin-bilirubin; *BCLC*, Barcelona Clinic Liver Cancer; *ECOG*, Eastern Cooperative Oncology Group; *RE*, radioembolization; *TACE*, transarterial chemoembolization

### Tumor response

In the overall cohort, 76 (42.9%) patients were responders, and in 136 (76.8%) of the patients, disease control was achieved (Table 2). The rate of objective response was significantly higher in the combination arm (61.6% [49.5–72.9%] vs. 29.8% [21.2–39.5%], *p* < 0.001), and a higher rate of disease control was seen in the combination arm (79.2% [73.0–91.2%] vs. 72.1% [62.4–80.4%], *p* = 0.075). Similarly, there were more patients with a complete response in the combination arm (13.7% vs. 3.8%, *p* = 0.022). The median DpR was also significantly higher in the combination arm (64.8% vs. 18.0%, *p* < 0.001).

**Table 2:** Response characteristics of treatment arms according to mRECIST

	Overall (*n* = 177)	RE + sorafenib (*n* = 73)	Sorafenib (*n* = 104)	*p*
Overall survival, months	14.3 (12.4–17.5)	15.0 (12.4–19.6)	13.8 (10.5–17.5)	0.77
Objective response, *N* (%)	76 (42.9)	45 (61.6)	31 (29.8)	** < 0.001**
Disease control, *N* (%)	136 (76.8)	61 (79.2)	75 (72.1)	0.075
Best response, *N* (%)				
• CR • PR • SD • PD	14 (7.9) 62 (35.0) 60 (33.9) 41 (23.1)	10 (13.7) 35 (47.9) 16 (21.9) 12 (16.4)	4 (3.8) 27 (26.0) 44 (42.3) 29 (27.9)	**0.022**
Time to response (months)	3.5 (3.6)	3.5 (3.8)	3.1 (3.3)	0.165
Percentage of DpR, median (mean)	36.6 (41.9)	64.8 (56.0)	18 (30.4)	** < 0.001**
ETS, *N* (%)	73 (41.2)	44 (60.2)	29 (27.8)	** < 0.001**
Time of DpR, months	4.1 (5.3)	4.9 (6.1)	3.9 (4.7)	0.065
Progression, *N* (%)	116 (65.5)	38 (52.0)	78 (75.0)	**0.001**
First progression site, *N* (%) (*n* = 116)				
• Liver • Extrahepatic • Both	91 (78.4) 13 (11.2) 12 (10.3)	25 (65.8) 6 (15.8) 7 (18.4)	66 (84.6) 7 (8.9) 5 (6.4)	0.02*
PFS, months	6.2 (4.9–7.8)	8.9 (6.3–9.9)	5.4 (4.1–7.4)	**0.022**

*CR*, complete response; *DpR*, depth of response; *ETS*, early tumor shrinkage; *PD*, progressive disease; *PFS*, progression-free survival; *PR*, partial response; *RE*, radioembolization; *SD*, stabile disease. *Liver vs. extrahepatic and both

**Fig. 2:**
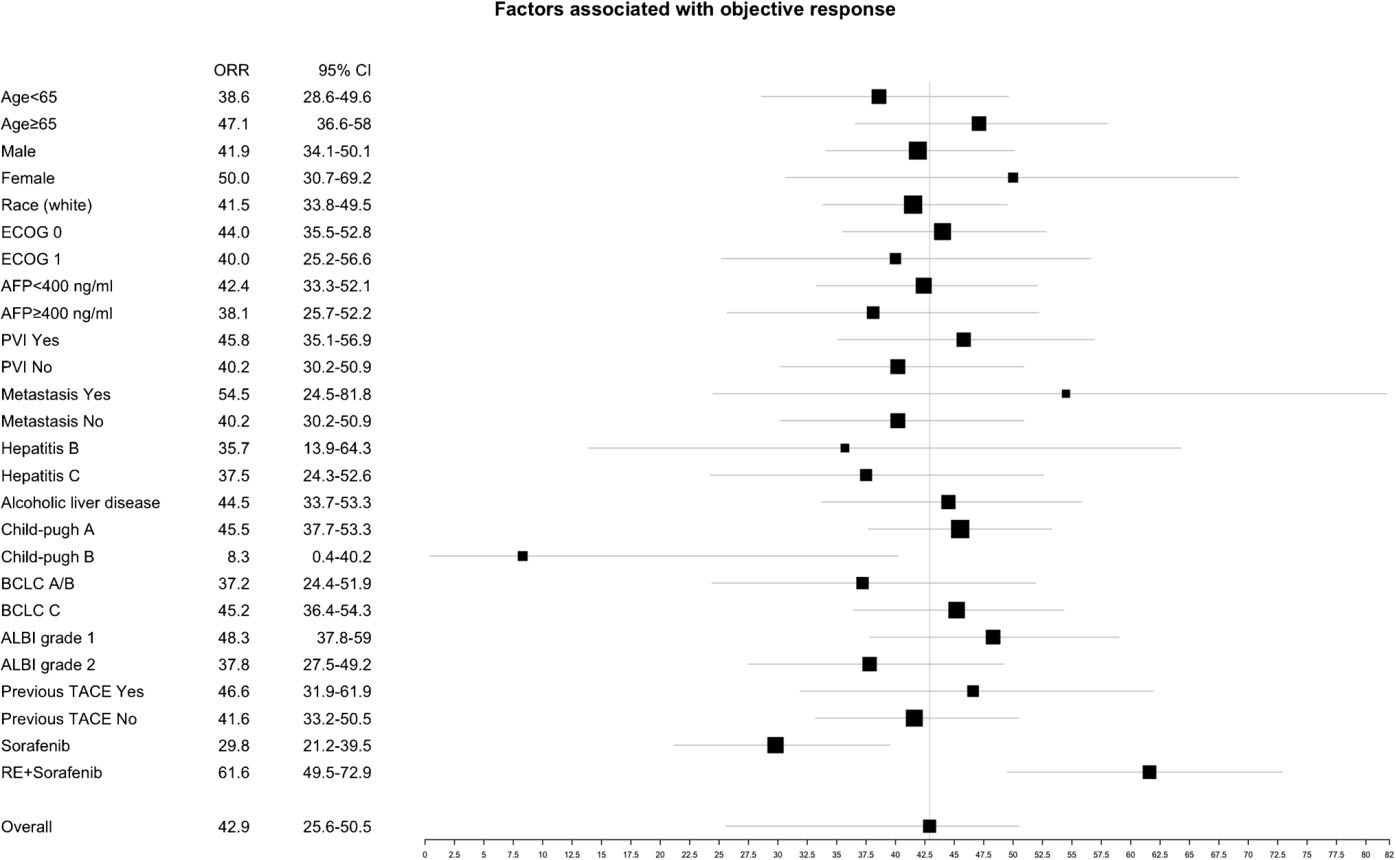
Factors associated with objective response. Objective response rate per mRECIST. AFP: alpha fetoprotein, ALBI: albumin-bilirubin score, BCLC: Barcelona clinic liver cancer, CI: confidence interval, ECOG: Eastern Cooperative Oncology Group, ORR: objective response rate, PVI: portal vein invasion, RE: radioembolization, TACE: transarterial chemoembolization

Except for the treatment arm, only the Child–Pugh B (*p* = 0.01) was significantly associated with lower objective response (Fig. 2).

The time to response was similar in both arms (3.5 months in combination and 3.1 months in sorafenib, *p* = 0.165).

Median time from randomization to the imaging used for ETS evaluation was 2.5 (range, 0.8–6.0) months. ETS was seen in 73 (41.2%) patients and was more common in combination arm (60.2% vs. 27.8%, *p* < 0.001).

### Disease progression

**Fig. 3:**
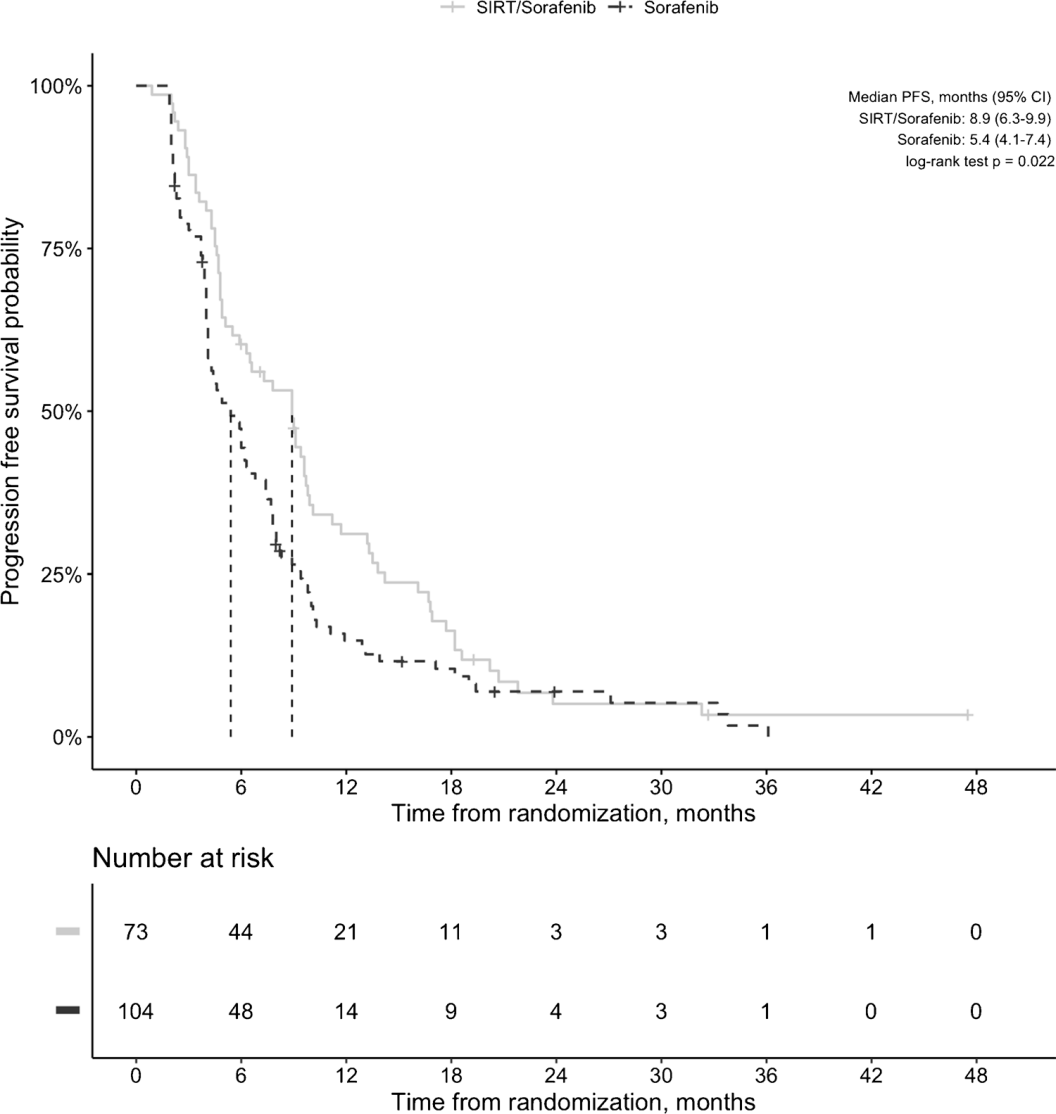
Progression-free survival of patients who received combination treatment compared to patients who received sorafenib only. CI, confidence interval; PFS, progression-free survival

During study period, 116 (65.5%) patients had progression, and more patients had progression in sorafenib arm (75% vs. 52.0%, *p* = 0.001). PFS was significantly longer in combination arm (8.9 [95% *CI*, 6.3–9.9] vs. 5.4 [95% *CI*, 4.1–7.4] months, *p* = 0.022; Fig. 3). Similarly, time-to-progression was significantly longer in the combination arm (10.1 [95% *CI*, 9.4–18.6] vs. 6.2 [95% *CI*, 4.9–8.0], *p* < 0.001). Also, the combination arm had longer hepatic PFS than the sorafenib arm (9.0 [6.3–10.1] vs. 5.7 [4.3–7.4] months, *p* = 0.014). Besides the treatment arm, Child–Pugh class B (< 0.001) was significantly associated with shorter PFS. Multivariate analysis confirmed that patients who received sorafenib monotherapy and Child–Pugh B were independent predictors of shorter progression-free survival (Table 3).

**Table 3:** Univariable and multivariable analysis of factors associated with progression-free survival

Parameter	Univariable analysis		Multivariable analysis	
	HR (95% *CI*)	*p* value	HR (95% *CI*)	*p* value
Treatment arm (sorafenib)	1.4 (1.1–2)	**0.022**	1.4 (1.02–2.0)	**0.025**
Sex (male)	0.74 (0.47–1.2)	0.2		
Age (≥ 65 years)	1.1 (0.78–1.5)	0.68		
ECOG (1 vs. 0)	1.1 (0.77–1.6)	0.58		
Cirrhosis (yes)	1.4 (0.87–2.2)	0.17		
Hepatitis B (yes)	1 (0.58–1.7)	0.98		
Hepatitis C (yes)	1.4 (0.98–2)	0.061		
Alcohol etiology (yes)	0.97 (0.71–1.3)	0.82		
TACE history (yes)	1.3 (0.94–1.9)	0.1		
PVI (yes)	1.1 (0.8–1.5)	0.550		
Child–Pugh (B)	3.5 (1.9–6.4)	** < 0.001**	2.6 (1.36–4.8)	** < 0.001**
BCLC (C)	1 (0.73–1.5)	0.81		
Outside up-to-7 criteria	1.3 (0.83–2)	0.26		
AFP (≥ 400 vs. < 400 ng/mL)	1.1 (0.76–1.5)	0.71		

*AFP*, alfa-fetoprotein; *BCLC*, Barcelona Clinic Liver Cancer; *ECOG*, Eastern Cooperative Oncology Group; *PVI*, portal vein invasion; *RE*, radioembolization; *TACE*, transarterial chemoembolization

First progression site was liver in 25 (65.8%) and 66 (84.6%), extrahepatic in 6 (15.8%) and 7 (8.9%), and both in 7 (18.4%) and 5 (6.4%) patients in combination and sorafenib arms, respectively. Progression in the liver was more common in the sorafenib arm (*p* = 0.02).

### Post-progression survival

In 116 patients with progression, the median post-progression survival (PPS) was 7.6 (6.2–9.0) months. PPS was 7.5 (6.0–11.5) months in the sorafenib arm and 7.9 (6.0–13.3) months in the combination arm (*p* = 0.86). Although the objective response was not correlated with PPS (*p* = 0.14), patients with disease control had significantly longer PPS than patients with the best response of progressive disease (9.0 vs. 3.4 months, *p* = 0.006).

## Discussion

Our results have shown that the combination of sorafenib with RE resulted in a higher response rate using mRECIST and a deeper and longer response than sorafenib monotherapy. Also, the progression rate was lower and time-to-progression was longer in the combination arm. Addition of RE to sorafenib treatment resulted in improved overall and hepatic PFS.

In addition to two negative trials that compared RE with sorafenib, in the SORAMIC study, the addition of RE to sorafenib has failed to improve overall survival in intermediate-advanced HCC patients compared to sorafenib treatment [12]. However, in the SARAH trial, RE resulted in better response and disease control rates, but not in longer PFS [10]. Also, in the treated population of the SIRveNIB trial, the objective response rate was higher, and PFS was longer in the RE arm [11]. In this substudy of the SORAMIC trial, a higher rate of objective response was seen in the combination arm. Furthermore, the addition of RE resulted in significantly longer PFS. These findings suggest an additive anticancer effect of RE to sorafenib treatment. This was also reflected with a higher rate of complete response in the combination arm (13.7% vs. 3.8%).

In SARAH and SIRveNIB, response assessment was done according to RECIST 1.1. Previous analyses showed a good correlation between RECIST and mRECIST in terms of progression only [24, 25]. Many previous studies have confirmed the better association between treatment outcome and response analysis according to mRECIST in HCC patients who received RE [17, 26, 27]. Recently, two studies, one in the Asian population and another in a Western population (a subanalysis of the sorafenib arm in SORAMIC), have shown that objective response assessment by mRECIST is able to predict survival after sorafenib treatment [19, 20]. Besides these, some additional imaging-based markers have been described for earlier detection of treatment response. ETS has been reported as an early predictor of a better outcome in HCC patients [22], and in our study, ETS was more common in the combination arm.

In addition to overall PFS, hepatic PFS was also shorter in the sorafenib arm. In the sorafenib arm, there were more patients with progression and progression in the liver as the first event. This was also seen in the SARAH trial, and there was a similar trend in SIRveNIB. However, combination therapy resulted in more prominent local disease control in the liver.

PPS was 7.5 months in the sorafenib arm and 7.9 months in the combination arm and 7.6 months in the study population. During the recruitment of the SORAMIC trial and also the other two trials, no second-line treatment was available. Within recent years, a number of systemic therapies have been shown to be effective in HCC patients in first-line and second-line for patients progressed under sorafenib [5, 13–15]. PPS in our study was similar to the survival of the placebo arm in the RESORCE trial (7.6 vs. 7.8 months) [13]. PPS was significantly longer in patients with initial disease control in our analysis. It may be speculated that effective second-line therapies could improve the survival in the combination arm, which had higher disease control (79.2% vs. 72.1%). This situation shows that the lack of an efficient therapy after progression might be one of the reasons for missed correlation between better response and longer survival in three RE trials.

Recently, atezolizumab-bevacizumab combination therapy has been shown to improve survival of patients with HCC compared to sorafenib and approved as the first-line therapy [5]. However, sorafenib is still used in the first line in cases where atezolizumab-bevacizumab was not available or contraindicated. Additionally, the efficiency of immune checkpoint inhibitors may be lower in patients with non-alcoholic steatohepatitis or Wnt/ß-catenin mutation [28]. Further on, it has been shown as superior to the atezolizumab-bevacizumab in terms of cost efficiency [29]. Also, updated results of the IMbrave 150 study showed that approximately 70% of the patients who received atezolizumab-bevacizumab within the trial had progression at the date of clinical cutoff [30]. Since this combination has not been shown to deteriorate liver functions, these patients are expected to be available for second-line therapies, and sorafenib is one of the two second-line therapies with lenvatinib [31]. However, best treatment sequence is not clearly defined yet. Our results show that combination of sorafenib and RE in selected cases might improve tumor control in those patients. Additionally, improvements in the RE technique, including better particle distribution via personalized dosimetry, improved the outcomes of RE in patients with HCC [32, 33]. These findings underline the need for re-definition of the exact role of RE in HCC again and ways to improve treatment sequencing after the failure or inefficiency of first-line therapies. Also, therapeutic synergism between radiation and immune checkpoint blockade has been suggested by preclinical studies, and a recent study showed 30.6% objective response according to RECIST after RE followed by nivolumab [34].

Considering the importance of liver function in the outcome of HCC patients, RE-induced liver disease has been described as deterioration in liver function at 4–8 weeks [35], and recent findings suggest RE may cause a delayed subclinical liver damage presenting with liver decompensation at 6 months [36]. Additionally, a sub-analysis of SORAMIC patients has shown that patients who received RE in addition to sorafenib had a higher increase in ALBI scores at 4 and 6 months compared to patients who received only sorafenib [37]. This might be the expense for the increased efficacy of combination therapy and the reason of missing translation of improved tumor control into better survival. Nevertheless, better patient selection and utilization of super-selective application of Y-90, instead of lobar approach, would translate into maintaining the liver function after radioembolization. Additionally, good tumor response might lead to downstaging in some patients and translate into the opportunity for potentially curative treatments including resection or transplantation [38, 39]. Furthermore, RE offers significant increase in metabolic function and size of the contralateral lobe [40]. One interesting finding in our study was lower response rates in Child–Pugh B patients. Only one of 12 Child–Pugh B patients had objective response. Although the exact mechanism behind this situation is not clear, it is probably related to higher treatment tolerability in patients with better liver function. Similar results have also been previously reported in HCC patients who received lenvatinib [41]. In that study, Child–Pugh B patients had lower relative dose intensity, and Child–Pugh class was significantly associated with objective response in multivariable analysis considering also the relative dose intensity. This possible relationship is also supported by the GIDEON study [42]. Despite the consistent overall safety profile across Child–Pugh classes, in Child–Pugh B patients, the median duration of sorafenib treatment was significantly shorter and adverse events leading to permanent discontinuation were more common compared to Child–Pugh A patients. In our study, Child–Pugh B was also associated with shorter PFS in multivariate analysis, similar to previous reports [43, 44].

This study has some limitations. First, only 61.4% of the PP population could be evaluated due to the patients underwent no follow-up imaging or were followed by ultrasound. This resulted in selecting a population with a longer OS compared to the trial population. However, the lack of efficient second-line therapies during the trial period was one of the reasons for a low rate of follow-up cross-sectional imaging. Second, there were minor baseline differences between treatment arms in this substudy, including more extrahepatic disease in the combination arm. But, a subgroup analysis of SORAMIC has shown that except for lung metastasis, the extrahepatic disease did not significantly lower treatment outcome [45]. There were more patients with ALBI grade 2 liver function in the sorafenib arm. However, there was no difference in the overall survival between treatment arms. Despite these limitations, this study comprises a cohort collected prospectively within a multicenter trial and only the patients treated strictly following the study protocol, and it represents the largest cohort in the literature showing the additional effect of RE on tumor response in patients receiving sorafenib.

In conclusion, our study showed that the addition of radioembolization resulted in better and deeper tumor control with improved objective response rates and progression-free survival in HCC patients receiving sorafenib.

## Data Availability

The datasets generated during and/or analyzed during the current study are available from the corresponding author on reasonable request.
